# Patellofemoral Instability in the Pediatric and Adolescent Population: From Causes to Treatments

**DOI:** 10.3390/children11101261

**Published:** 2024-10-18

**Authors:** Anthony Ricciuti, Katelyn Colosi, Kevin Fitzsimmons, Matthew Brown

**Affiliations:** Connecticut Children’s Sports Medicine, 399 Farmington Ave., Farmington, CT 06032, USA; aricciuti@connecticutchildrens.org (A.R.); kcolosi@connecticutchildrens.org (K.C.); kfitzsimmons@connecticutchildrens.org (K.F.)

**Keywords:** patellar dislocation, pediatric, adolescent, medial patellofemoral ligament (MPFL), tibial tubercle to trochlear groove (TT–TG), trochleoplasty, trochlear dysplasia

## Abstract

Background: Patella instability is one of the most common knee injuries in the adolescent patient. There are several pathoanatomic risk factors which should be assessed via several modalities, including X-rays, magnetic resonance imaging (MRI), or even CT scan. Objectives: We intend to review these risk factors along with the nonsurgical and surgical techniques used to prevent recurrent dislocations. Methods: We completed an extensive review of the recent literature concerning pediatric and adolescent patellar dislocation and subsequent treatment modalities. Results: We review in detail the risk factors such as patella alta, trochlear dysplasia, lateralization of the tibial tubercle or medialization of the trochlear groove (increased tibial tubercle to trochlear groove (TT–TG) distance), lower limb malalignment, excessive femoral anteversion and/or tibial torsion, and hyperlaxity. There are classification systems for dislocators, and a natural progression of instability that patients often proceed through. Only after a patient has continued to dislocate after bracing and physical therapy is surgical treatment considered. Surgical techniques vary, with the workhorse being the medial patellofemoral ligament (MPFL) reconstruction. However, there are a variety of other techniques which add onto this procedure to address other anatomic risk factors. These include the tibial tubercle osteotomy to address a large TT–TG distance or trochleoplasty to address the lack of a trochlear groove. Conclusions: Nonsurgical and surgical treatments for patella dislocators are tailored to the pathoanatomic risk factors in each patient.

## 1. Introduction

Lateral dislocation of the patella is a common injury in children. It can occur in previously healthy and anatomically normal knees, but there are several abnormalities that can predispose to patellar instability. The mainstay of treatment for an initial patellar dislocation is nonsurgical; however, treatment often progresses to surgical interventions. Recently, surgeries have come to focus on addressing the individual anatomic predispositions to dislocation. In this paper, we focus on individually documenting each anatomic risk factor and the respective surgical interventions, with a particular focus on addressing the trochleoplasty versus tibial tubercle osteotomy debate.

## 2. Epidemiology

The annual incidence of first-time patellar dislocation is estimated to be between 23.2 and 108 per 100,000 person-years [1,2,3], making this knee injury one of the most prevalent in the adolescent age group. Several recent reports suggest that the rates of dislocation and its subsequent surgical treatments have been increasing [4,5]. However, the surgical increase may be due to either an increase in surgical management or to an increase in patellar dislocations itself [6].

The majority of cases of acute traumatic patellar dislocations are associated with athletic activity in adolescents [7]. While the relative risk of different sporting activities varies, the average dislocation rate is 1.95 per 100,000 athletic exposures (AE). The sports with the highest number of dislocations differ by sex, with gymnastics (6.19/100,000 AE) most prevalent in females and football (4.10/100,000 AE) in males [8].

Historical data suggest that males and females have the same propensity for patellar dislocations early in childhood, though this changes during adolescence, when females seem to have a higher risk for first-time dislocation [1,2,9]. This change is multifactorial, involving such anatomical differences between sexes as greater Q angle and an increase in ligamentous laxity. Competitive female athletes have a greater propensity to non-traumatic patellar dislocation as opposed to their male counterparts, who have far more traumatic dislocations [8].

## 3. Pathoanatomic Factors

Several pathoanatomic risk factors for patellar instability have been well established and should be evaluated in patients presenting with patellar instability. These include patella alta, trochlear dysplasia, and lateralization of the tibial tubercle or medialization of the trochlear groove (increased tibial tubercle to trochlear groove (TT–TG) distance). We also evaluate lower limb malalignment, excessive femoral anteversion and/or tibial torsion, hyperlaxity, and any syndromic associations.

### 3.1. Medial Patellofemoral Ligament

**Figure 1:**
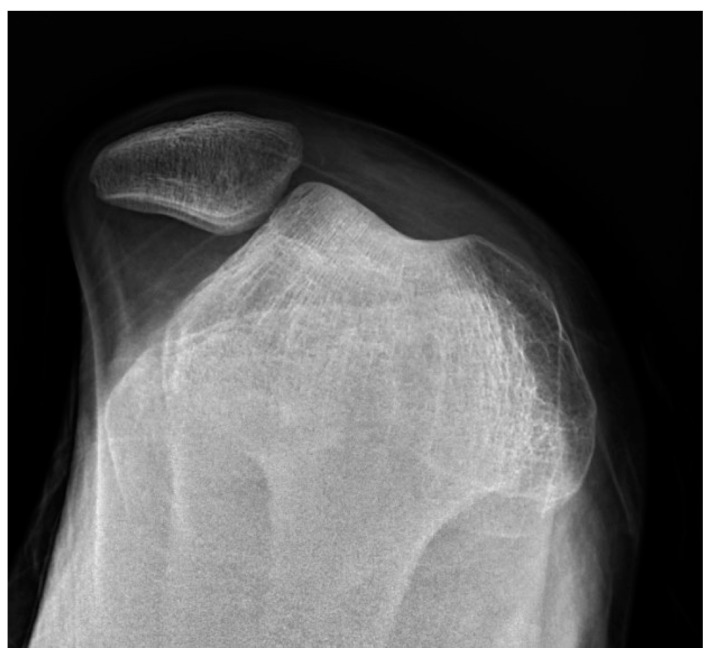
A 15-year-old with lateral patellar dislocation.

Accepted patellofemoral mechanics recognize that the patella is guided into the trochlea by the MPFL during knee flexion from a fully extended position. After the knee reaches approximately 15 degrees of flexion, the lateral portion of the trochlea takes over as the primary stability factor of the patella [10]. The MPFL is recognized as the most important stabilizer of the patella. An insufficient MPFL may reduce the necessary force resulting in patellar dislocation by 50% when the knee is extended [11]. Isometry of the medial patellofemoral ligament has been considered by several groups. Stevens et al. concluded that the MPFL was most isometric when positioned at the midpoint between the medial epicondyle and the adductor tubercle [12]. Changes in attachment by as little as 5 mm proximal or distal resulted in significant changes in length of the MPFL. Mulliez et al. found MFPL isometry varies depending upon the portion of MPFL tested and the position of the knee from extension to 30 degrees of flexion [13]. The medial patellofemoral ligament, as the primary restraint resisting lateral patellar dislocation, has to be sprained or torn after a known dislocation (Figure 1).

### 3.2. Patella Alta

**Figure 2:**
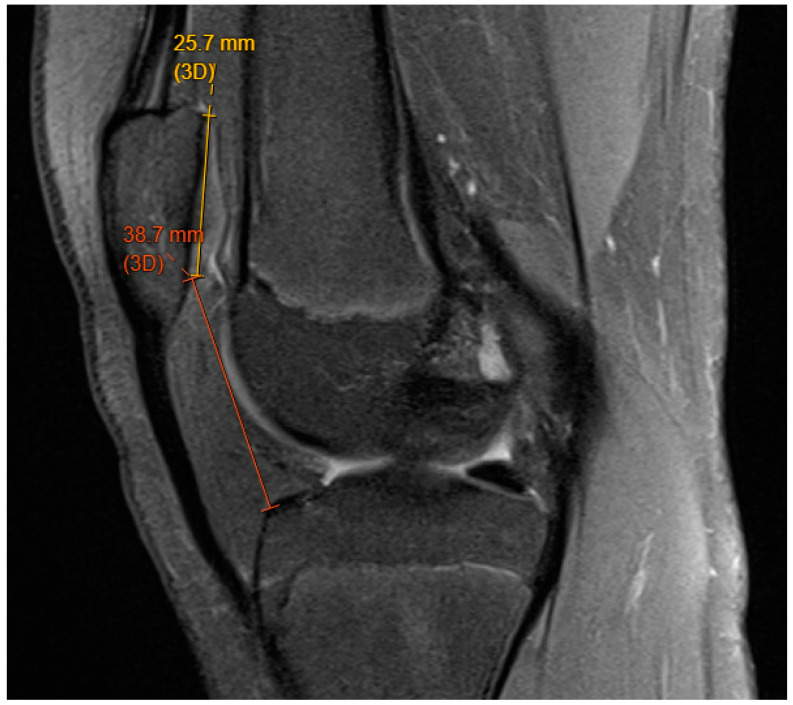
Canton Deschamps ratio of 1.5 (38.7/25.7) seen in 14-year-old chronic patellar dislocator.

A patella more superior in relation to the trochlear groove is at increased risk for patellar dislocation. The patella remains proximal to the trochlear groove with the knee in full extension and through early flexion, meaning that the patella has to cover a longer arc of motion to reach the stability of the groove. There are several measurements to calculate patella alta, including the Canton-Deschamps ratio [14]. This measurement on lateral radiograph evaluates the ratio between patellar length and patellar distance from the tibial plateau. with a ratio greater than 1.2 considered abnormal (Figure 2).

### 3.3. Trochlear Dysplasia

While a normal trochlea serves as a groove for the patella to travel within, dysplasia lends to an abnormal depth and alignment. Dejour et al. categorized trochlear dysplasia into four grades based on XR lateral imaging and axial cuts of a CT or MRI scan [15]. In brief, a type A trochlea has a shallow groove, type B has a flat trochlea, type C is a convex trochlea, and type D has a convex trochlea with a hypoplastic medial femoral condyle. Numerous measurements have emerged to delineate and differentiate trochlear dysplasia, including sulcus angle, lateral trochlear inclination, and ventral trochlear prominence [16]. There has been extensive investigation evaluating different variations of trochlear dysplasia, as it has been cited as the greatest anatomic risk factor for recurrent patellar dislocation [17].

**Figure 3:**
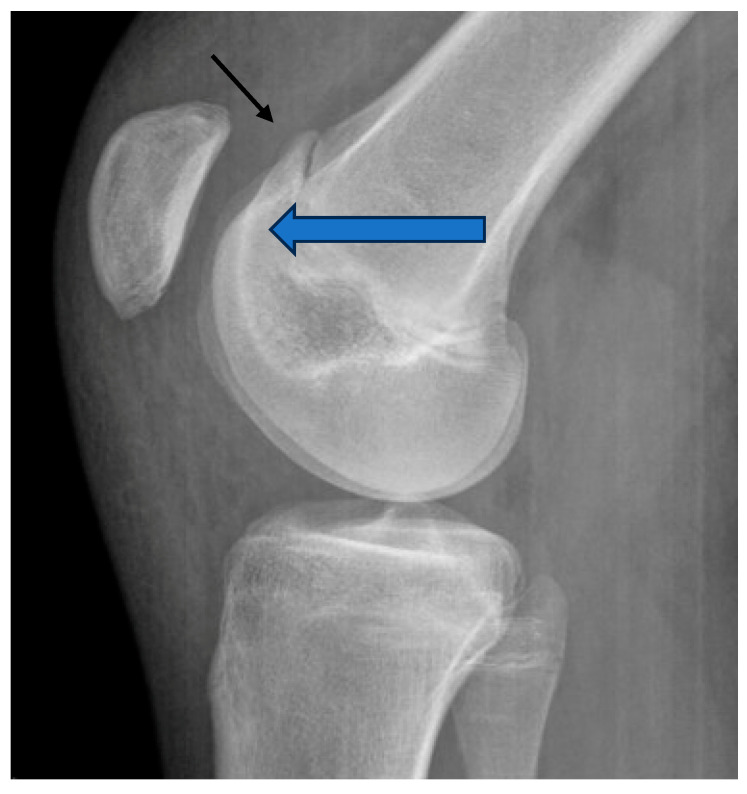
Crossing sign (bold arrow) and supratrochlear spur (thin arrow) seen in lateral XR of 15-year-old patellar dislocator.

Identifying patients with symptomatic trochlear dysplasia that are at risk for recurrent patellar instability and early patellofemoral joint (PFJ) osteoarthritis (OA) is crucial in addressing this common cause of patellofemoral pain and instability and reducing the risk for future OA. Diagnosis of trochlear dysplasia in the patient with patellar instability or patellofemoral pain can be made on radiographs. The crossing sign and trochlear bump sign are two common radiographic findings that can be identified on standard knee radiographs. The crossing sign is the convergence of the radiographic lines with the proximal trochlea and anterior femur [18]. When the line corresponding to the trochlea groove crosses the anterior lines of the medial and lateral condyle, this corresponds to a shallow proximal trochlea without a groove for the patella to engage in [18]. The trochlear bump sign corresponds to a proximal trochlear spur (Figure 3). The anterior cortex of the distal femur should remain flat when it reaches the proximal trochlea. Any bone seen anterior to a line drawn down the anterior femoral cortex on lateral radiograph corresponds to a supratrochlear spur seen in Dejour types B and D trochlear dysplasia [18].

**Figure 4:**
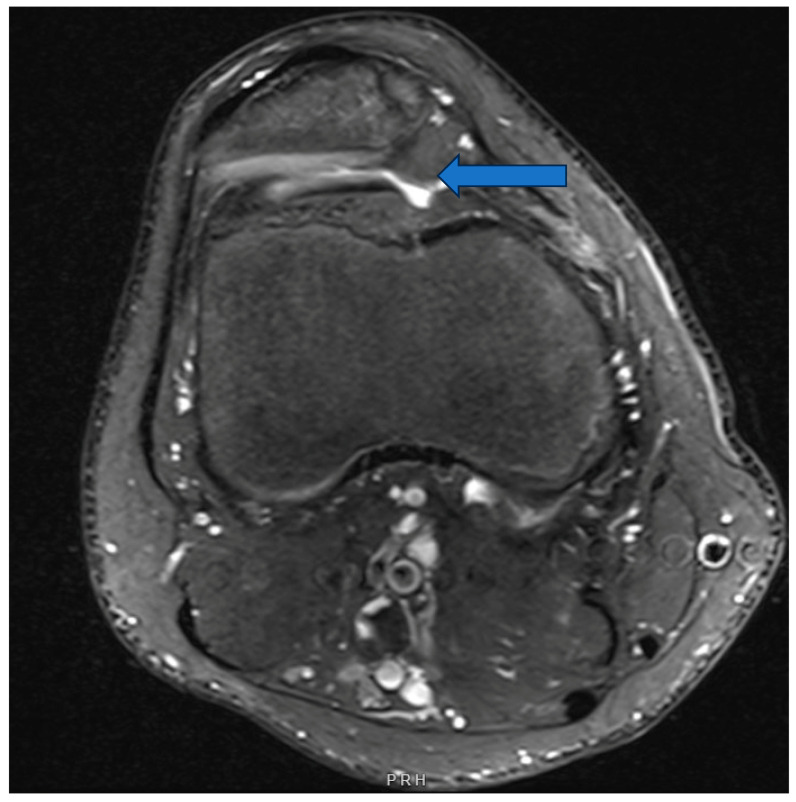
A15-year-old male with lack of a trochlear groove (arrow points to convex lateral femoral condyle, no groove noted), with laterally subluxated patella.

On axial MRI imaging, the depth of the trochlear groove and presence of shallow, flat or convex proximal trochlea can be measured by lateral trochlear inclination angle (LTI) (Figure 4). The two-image LTI uses the proximal trochlea and a more distal and clear view of the posterior femoral condyles to assess the severity of trochlear dysplasia [19].

### 3.4. Elevated Tibial Tubercle to Trochlear Groove Distance (TT–TG)

The TT–TG distance is measured on axial cuts of an MRI or CT scan, and in the pediatric and adolescent population, the accepted values change along with age and relative maturity. The tibial tubercle to trochlear groove (TT–TG) distance is defined as the distance separating the perceived center of the trochlear groove on MRI or CT and the midpoint of the tibial tubercle in the axial plane [20]. A clinically relevant TT–TG distance has been defined as greater than or equal to 20 mm in adults, because this has a greater than 90% association with patellar instability [21]. This measurement is far more relevant for adult patients then the pediatric population because TT–TG distances, as they relate to patellar instability, are dependent on chronologic age. Dickens et al. demonstrated that pediatric patients with patellar instability had an average TT–TG distance of 12.1 mm [22]. The value of TT–TG distance can vary based on the degree of knee flexion on cross-sectional imaging, as well as physeal maturity. This provides limited utility for determining anatomic malformation location [23].

### 3.5. Lower Limb Malalignment

**Figure 5:**
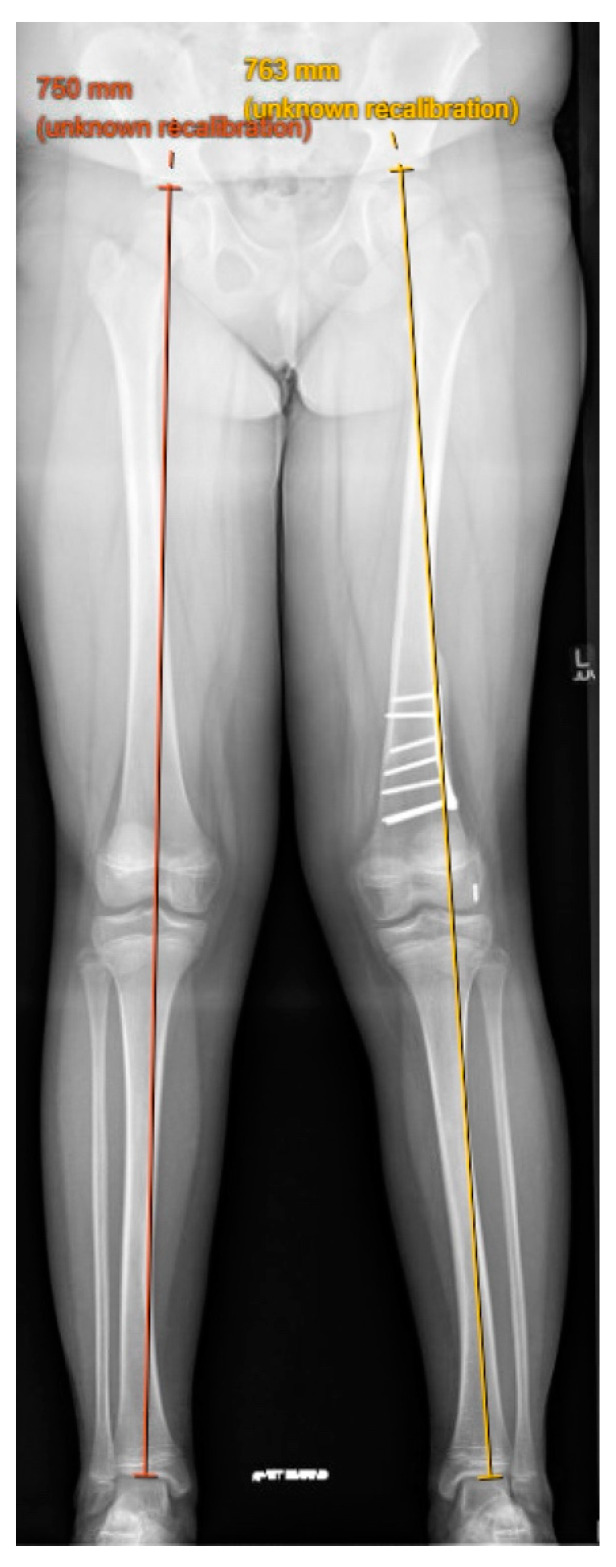
An 11-year-old female with left knee valgus and patellar instability after previous femoral derotation osteotomy and MPFL reconstruction for chronic patellar dislocations.

Q-angle represents the resultant force vector of the quadriceps and patellar tendons acting on the patella. The Q-angle is measured by drawing a line through the center of the patella to the anterior superior iliac spine and another from the tibial tubercle through the patella center. The intersection of these two lines is the Q-angle; the normal value for this angle is 13 to 18 degrees [24]. Valgus malalignment at the knee can lead to patellar dislocation due to an increased Q-angle. Patients aged 12 or older should not have genu varum or valgum greater than 8 degrees; however, there is no numerical definition of pathologic valgus. Parikh et al. suggested that guided growth of distal femur be performed for correction of genu valgum in the presence of patellar instability when the lateral distal femoral angle was 84 degrees or less and the mechanical axis was in the lateral compartment on weightbearing mechanical axis XR (Figure 5) [24].

### 3.6. Femoral Anteversion and Tibial Torsion

Miserable malalignment is a term used to refer to the combination of femoral anteversion and tibial torsion, occasionally combined with genu valgum. Both femoral anteversion and external tibial torsion increase lateral pressures on the patella, causing increased medial patellofemoral ligament (MPFL) strain along with lateral patellofemoral contact pressure. Takagi et al. demonstrated that during weight bearing, external tibial torsion and femoral anteversion (alignment parameters in the transverse plane) are most associated with recurrent patellar dislocations [25].

### 3.7. Hyperlaxity

Increased tissue elasticity leads to increased joint mobility, of which Ehlers–Danlos (ED) syndrome is the syndrome most commonly associated with patellar dislocation. It has been demonstrated that ED patients have an increased risk of dislocation due to hyperlaxity [26]. The physical exam evaluation for hyperlaxity is the Beighton score, of which 5 of 9 is positive for adults, and 7 of 9 has been suggested for positivity in children [26].

### 3.8. Syndromes

Several syndromes have been associated with patellar dislocations, including nail–patella syndrome, Marfan’s Syndrome and Down’s Syndrome. Each of these syndromes is approached on an individual basis, as children with different diagnoses have varied functional levels and needs [27]. There are not much data in the literature concerning these patients, especially since they can be the youngest at presentation and more difficult to treat appropriately.

## 4. Classification of Dislocations

Several differing classification systems exist for patellar dislocations, each with a different focus. Parikh and Lykissas focused on typifying patellar dislocations, with the types being (1) first-time, (2) recurrent, (3) dislocatable, and (4) dislocated, along with voluntary and syndromic dislocations [28]. Chotel et al. focused more on the pediatric anatomical, pathophysiologic, biomechanical, and clinical factors at play in patellar dislocations and how these related to potential surgical treatments [29]. Schlichte et al. also developed a grading system of patellar dislocations, which delineates four types with specific features: (1) syndromic—connective tissue disorders (hyperlaxity), (2) obligatory—dislocates every knee flexion moment, (3) fixed—irreducible, (4) traumatic—first time vs. recurrent [30].

Traumatic dislocations in the acute setting lead to a disruption of the MPFL. This often leads to subsequent dislocations requiring less force, due to insufficiency of medial structures. Irreducible patellar dislocations are the rarest type but can be seen most often with congenital limb deformity. The majority of obligatory dislocations occur with the knee moving into flexion, with reduction occurring with subsequent extension. Finally, syndromic dislocators have a genetic condition which predisposes them to patellar dislocations due to reasons including anatomy and hyperlaxity. They can be more vulnerable to fixed or frequent patellar dislocations.

## 5. The Natural Progression of Patellar Dislocations

The progression of patellar dislocations varies due to a number of factors like patient anatomy, activity level, and age. The incidence of repeat dislocation has been reported between 15–75% [3,14,31,32,33,34]. A patient may have an isolated dislocation or a progression to recurrent subluxations or full chronic dislocations. There are numerous bilateral knee anatomic relationships which predispose the contralateral knee to the same instability as the presenting knee [35], though the contralateral knee tends to dislocate at a much lower rate (5–8%) [3,21]. It has been suggested that this is due to the patellofemoral morphological differences between the ipsilateral, dislocatable side and the contralateral side [36].

The consequences of recurrent dislocation are numerous, including: persistent pain, mechanical symptoms, osseous or chondral damage, loose body, and an inability to return to sport. The presentation or inclusion of each of these variables depends on many factors, including the initial inciting event, underlying pathoanatomy, ligamentous laxity, activity level, and previous treatment course. The incidence of osseous or chondral damage varies greatly based on underlying anatomy and is best assessed with magnetic resonance imaging (MRI). The most common lesions seen as sequelae on MRI are in the medial patellar facet and the lateral femoral condyle, which can been seen in 70–96% of patients [37].

## 6. Assessing Patellar Dislocation Risk

Several factors conspire to increase the risk of first-time or recurrent patellar dislocations. Demographically, younger age and open physes increase the overall risk, as do anatomical factors such as trochlear dysplasia and patella alta [7,14,21]. As a patient accumulates several different risk factors, the overall re-dislocation probability increases, reaching as high as 88% [14,21]. The most influential factors, per Lewallan et al., are open physes and trochlear dysplasia, with a patient having both of these factors presenting with a 68.8% chance of recurrent dislocation [7]. This was three times higher than the recurrent dislocation rate of a skeletally mature patient without trochlear dysplasia.

Family history of patellar instability has been shown to be a risk factor for patellar dislocation. This has been demonstrated in both an increased risk of contralateral dislocation and recurrent dislocation [38]. In the adolescent cohort, the risk of recurrent dislocation after an initial event has been shown to vary from 15 to 54%, with Zhang et al. demonstrating a pooled rate of 41.8% [39]. The risk of a second dislocation peaks during adolescence, with the first five years after the initial dislocation the time of highest risk [1,7]. A number of studies have attempted to develop a predictive model so that high-risk patients may be identified earlier; however, none of these have been validated yet [40,41,42].

## 7. Nonsurgical Interventions

A significant portion of the literature has studied operative versus nonoperative approaches to treating first-time dislocations [43,44]. It is common practice that unless there are loose osteochondral fragments within the joint as a sequela of the patellar dislocation/reduction process, nonoperative treatment can be entertained. This usually consists of rest, ice, non-steroidal anti-inflammatories, bracing, and physical therapy. The use of a specific patella-stabilization type of brace can help to increase patellar stability and overall joint mechanics [45]. Formal physical therapy begins either after the receipt of an MRI without osteochondral loose bodies or when the patient is initially seen in clinic if they are asymptomatic without a knee effusion. Primary goals in physical therapy focus on restoration of range of motion, normalization of gait, and restoration of knee extension strength. Progression to sport-specific rehabilitation follows with an anticipated return to sport roughly 12 weeks after the injury. For the first 12 months after the initial dislocation, a patella-stabilizing brace is used for sport, and after that at the patient’s discretion.

## 8. Surgical Interventions

### 8.1. Medial Patellofemoral Ligament Reconstruction (MPFLR)

A medial patellofemoral ligament reconstruction is the standard of care for recurrent dislocators who have failed non-operative modalities or as part of a larger surgical plan for a first-time dislocator with intra-articular loose bodies. It has been demonstrated that, in the younger pediatric and adolescent populations, use of a gracilis allograft for reconstruction can be superior to autograft [46]. Recurrence following MPFL reconstruction has been reported to be less than 3% [47]. Non-surgical management is generally accepted as standard for first-time dislocations without associated loose bodies or fractures. Individuals with trochlear dysplasia may be directed toward surgical intervention to reduce risk of recurrent dislocation; however, this remains open for debate.

If a patella is congenitally subluxed laterally or is unable to be translated medially to the trochlear groove, a lateral lengthening may be performed [48]. However, this is a graduated lengthening without complete open or arthroscopic release. While there are viable data on the benefits of performing a lateral lengthening in addition to an MPFL [48], a number of articles have demonstrated that a release of lateral structures, either alone or in conjunction with an MPFL reconstruction, can destabilize the patellofemoral joint and increase the risk of dislocation [49].

### 8.2. Elevated Tibial Tubercle to Trochlear Groove Distance

Indications and the role for tibial tubercle osteotomy have undergone change as other surgical treatments for patellar instability have become more widely studied and reproducible. The aforementioned TT–TG measurement is notable, as increased distances predispose patients to patellar instability, both when identified in conjunction with or without significant trochlear dysplasia [22]. When it has been determined that a skeletally mature patient’s patellar maltracking is primarily associated with an abnormal TT–TG distance and conservative management has failed, a patient may be indicated for a tibial tubercle osteotomy [50].

The anteromedialization tibial tubercle osteotomy (TTO) has been a workhorse for treating patients with recurrent patellar instability and an elevated tibial tubercle-trochlear groove distance (TT–TG). In this surgery, a relatively thin wafer of the tubercle is elevated and then translated antero-medially and secured using screws [51]. This procedure targets a lateralized tibial tubercle and the associated patellar maltracking, but with proper surgical planning, the osteotomy can be multiplanar in nature and be utilized to address the aforementioned maladies, such as performing osteotomies for distalization [52]. The role of an isolated TTO as the primary method to prevent recurrent lateral patellar dislocation has lessened, while it continues to be used in conjunction with other procedures. Often paired with MPFL reconstruction, this procedure has since remained a well-established and popular surgical treatment for patellar instability [53]. Indications for TTO include closed physes, recurrent lateral patellar dislocations, TT–TG > 20 mm, and patella alta. Contraindications for TTO include patella baja, open physes, and advanced patellofemoral joint arthritis.

Outcomes have historically proven that when indicated, tibial tubercle osteotomies are an effective surgical intervention for patellar instability patients, especially when paired with MPFL reconstructions, with low recurrent instability rates [54]. This is also true with syndromic patients, as a combined MPFL-TTO has been shown effective in short-term follow-up [55]. As noted with all patients with patellar instability and patellofemoral disorders, surgical decision-making is multifactorial—the decision to proceed with a tibial tubercle osteotomy does not necessitate that a patient undergo medialization. Despite an elevated TT–TG, patients may not have a lateralized tibial tubercle, and if this is the case, proceeding with a medialization may result in poor clinical outcomes and cartilage breakdown [54]. Complications of TTO include skin necrosis, osteotomy nonunion, symptomatic hardware, and tibia fracture. The risk of skin necrosis is much less with distalizing and anteromedializing techniques, but still present.

### 8.3. Trochlear Dysplasia

The most common pathoanatomical risk factor for patellar instability is trochlear dysplasia [56]. Trochlear dysplasia is an abnormally shaped trochlear groove that can either be shallow, flat, or in more severe cases, convex, which can be a source of patellar instability. Trochlear dysplasia is found in 85% of patients with recurrent patellar instability. [21]. The relationship between a normally shaped trochlea and patella is integral in reducing contact pressure, increasing contact area, improving lateral tilt of the patella, and improving stability. With trochlear dysplasia, there is increased patellofemoral contact pressure, and with reduced congruency of the patellofemoral joint (PFJ), there is decreased contact area, which may predispose patients with trochlear dysplasia to PFJ OA [57].

A normal patella engages in the proximal trochlea at 20° of knee flexion, and then remains centered in the trochlea through the remainder of knee flexion [58]. The MPFL is the main restraint to lateral patellar translation in the first 20° of knee flexion. However, when there is a shallow proximal trochlea, or more severely, convex trochlea, the force required to dislocate the patella is less in extension. The force needed to dislocate the patella in shallow proximal trochlear grooves mimics that of knees with incompetent MPFLs [59]. This underscores the importance of bony congruency of the patellofemoral joint in the role of patellar stability.

With trochlear dysplasia being the most common anatomic risk factor for patellar instability, treatments have begun to focus on the dysplastic trochlea directly. Surgical techniques have improved over the past 10–15 years and have demonstrated a reduction in complications, improved patient-reported outcomes, and higher return to sports rates. The complication profile and rates of trochleoplasty are now similar to those of other patellar stabilizing procedures.

Diduch et al. advise that surgical indications for trochleoplasty should include patients with severe dysplasia that have a convex proximal trochlea with or without a supratrochlear spur. These indications include patients with Dejour Type B and D trochlear dysplasia. When other anatomic risk factors are accounted for, trochleoplasty for patients with high-grade trochlear dysplasia is a powerful procedure to restore the anatomy of the patellofemoral joint, to improve congruency and stability, and to reduce the risk of future instability events [60].

Current surgical treatment of trochlear dysplasia focuses on the sulcus deepening trochleoplasty procedure, where the proximal trochlear articular cartilage is elevated and a new and deeper trochlear groove is fashioned. The previously hypoplastic and medial trochlear groove is also moved laterally to restore the anatomy and mechanics of the patellofemoral joint. At the time of sulcus deepening trochleoplasty, the supratrochlear spur is also removed [61].

Outcomes of sulcus deepening trochleoplasty in the recent literature are promising. The prior complication rates were associated with trochleoplasty performed using a different technique [62]. The current literature centers on the more commonly performed Bereiter technique along with deepening trochleoplasty, the Dejour technique, or arthroscopic trochleoplasty [61]. In a review of complications associated with trochleoplasty, redislocation rate, return to the operating room, hardware failure, and fracture were evaluated [63]. Meta-analysis demonstrated redislocation rates after the Bereiter technique of 0.02–0.06% and 0.09% after the Dejour technique, with complication rates of 0.08% and 0.02%, respectively. Currently, trochleoplasty has equal or lower overall complication rates than other patellar stabilizing procedures, including MPFL reconstruction (4.1%) and medializing tibial tubercle osteotomy (18%) [64]. Trochlear dysplasia is the most common anatomic risk factor for patellar instability, and modern techniques allow for safe and reliable creation of a trochlear groove to reduce the risk of recurrent instability and pain and improve patient function.

### 8.4. Patella Alta

A patella that is situated higher-than-normal in relation to the trochlear groove leads not only to an increase in perceived patellar instability but also to a decrease in patellofemoral contact area and overall stress at the joint [65]. For adults, the solution is to distalize the tibial tubercle, pulling the patella inferiorly into the groove in the femur [66]. However, for children with open physes, this is not an option. A number of options have been investigated in this age group, including partial or complete distal transposition of patellar tendon, patellar tendon imbrication and patellar tendon shortening methods [67]. However, excellent results have been found from performing an isolated MPFL reconstruction, and there is evidence to suggest this also reduces the patella alta initially seen [68].

### 8.5. Genu Valgum

The presence of genu valgum or “knock-knees” has been associated with patellar instability due to the increase in lateral forces exercised on the patellofemoral joint. This is typically surgically resolved using an osteotomy in adult patients; however, in children and adolescents with open growth plates, guided growth can be successfully performed [69]. It has been demonstrated that an MPFL reconstruction and guided growth can be successfully performed in the same patient sequentially [24].

### 8.6. Femoral Anteversion

Rotational malalignment has been recognized as a risk factor in patellofemoral instability [70]; however, it is much more difficult to address in an adolescent with a simple procedure. It has been shown that patients undergoing an MPFL and tibial tubercle osteotomy have worse outcomes when they have underlying femoral anteversion [71]. When the diagnosis of femoral anteversion appears to be a major driver of patellar instability, a femoral derotation osteotomy is justified [72]. It appears that the source of femoral anteversion may vary by person, suggesting that an individualized approach to osteotomies (proximal vs. shaft vs. distal) may be appropriate [73].

### 8.7. Quadricepsplasty

Patients with either a fixed lateral patellar dislocation or an obligatory (habitual) dislocation can present with an array of anatomical abnormalities, including short extensor mechanism and tight lateral structures that lead to patellar dislocation [74]. A fixed dislocation is where the patella remains constitutively dislocated throughout the knee range of motion. A habitual patellar dislocation occurs when the patella dislocates every time the knee flexes, and both types may lead to future OA.

There is no standardized treatment algorithm for either fixed or habitual patellar dislocation. Surgical treatment factors in the varied etiologies of these conditions, in addition to the presence of open physes. In general, MPFL reconstruction is not sufficient to remove the deforming forces on the patella, so concomitant procedures are required. Often, this starts with an extensive lateral retinacular release, extending into the vastus lateralis tendon and even involving a lengthening of the vastus lateralis tendon itself. If, after these procedures, the patella continues to dislocate laterally when the knee is flexed, then a formal quadriceps Z-lengthening is performed to address the shortened extensor mechanisms while maintaining the patella in the trochlear groove during flexion.

Historically, there are several procedures that have attempted to lengthen the quadriceps mechanism and re-align the patella in order to manage habitual or fixed patellar dislocation. A number of groups have promoted a combination of procedures, including a Roux-Goldthwait distal patellar tendon realignment, lateral release, medial plication, Galeazzi procedure, and/or vastus medialis advancement. More recently, there have been good results reported by Ellsworth et al., who are performing a gradual extensive lateral release or lengthening, vastus lateralis lengthening, and a separate Z-lengthening of the rectus and intermedius. The surgeon is able to preferentially lengthen the lateral aspect of the quadriceps tendon more than the medial aspect [75].

## 9. Summary

The management of patellar instability in the pediatric and adolescent population continues to evolve, both in terms of new procedures but also improvements to existing methods [76]. A number of factors must be taken into account when the decision for surgical intervention is made; these include physeal status, anatomic factors, and patient request. The workhorse surgery has become the MPFL reconstruction, with additions including tibial tubercle osteotomy, trochleoplasty, and lateral lengthening, depending on patient-specific factors.
